# A discursive review of the textual use of ‘trapped’ in environmental migration studies: The conceptual birth and troubled teenage years of trapped populations

**DOI:** 10.1007/s13280-017-1007-6

**Published:** 2018-02-12

**Authors:** Sonja Ayeb-Karlsson, Christopher D. Smith, Dominic Kniveton

**Affiliations:** 1 University of Sussex, Chichester I, Brighton, BN1 9QJ UK; 2 United Nations University Institute for Environment and Human Security (UNU-EHS), Platz der Vereinten Nationen 1, 531 13 Bonn, Germany; 3 University of Sussex, Arts C, Brighton, BN1 9SJ UK

**Keywords:** Climate change, Critical Discourse Analysis, Environmental migration, Immobility, Textual analysis, Trapped Populations

## Abstract

First mooted in 2011, the concept of Trapped Populations referring to people unable to move from environmentally high-risk areas broadened the study of human responses to environmental change. While a seemingly straightforward concept, the underlying discourses around the reasons for being ‘trapped’, and the language describing the concept have profound influences on the way in which policy and practice approaches the needs of populations at risk from environmental stresses and shocks. In this article, we apply a Critical Discourse Analysis to the academic literature on the subject to reveal some of the assumptions implicit within discussing ‘trapped’ populations. The analysis reveals a dominant school of thought that assisted migration, relocation, and resettlement in the face of climate change are potentially effective adaptation strategies along a gradient of migrant agency and governance.

**Electronic supplementary material:**

The online version of this article (10.1007/s13280-017-1007-6) contains supplementary material, which is available to authorized users.

## Introduction

Numerous references to Trapped Populations^1^ have emerged since the concept’s recent arrival within migration studies. As a lens through which to identify those people most affected by climate change, the notion of being ‘trapped’ is potentially useful to expose the social inequalities of impacts and variations in coping or adaptive capacity. However, considerable ambiguity surrounds both the foundation of the concept and the normative implications of its use. A shortage of critical analysis means that in most instances vagueness serves to disguise any precise determinations of who may be ‘trapped’ and what they may be trapped by. Humanitarian efforts intended to provide support to involuntarily immobile people may therefore risk being ineffective or imposing externally formed ideals surrounding mobility onto vulnerable populations.

Notions of involuntary immobility (e.g. Carling 2002; Lubkemann 2008) and references to peoples’ inability to escape environmentally risky and vulnerable locations (e.g. Blaikie et al. 1994; Thiede and Brown 2013) have existed in the literature on environmental migration for some time. However, the ground-breaking UK Government’s Foresight Migration and Global Environmental Change (MGEC) report (Foresight 2011) was first to identify such people as Trapped Populations. In doing so, the report recognised the complex relationships between human activity and the environment, while suggesting that impoverished people may end up ‘trapped’ at the hands of a double set of risks that render them not only more vulnerable to environmental threats, but also less able to escape or move away from them. A trilogy of potential mobility outcomes resulting from environmental change was proposed which distinguished between migration, displacement, and immobility.

Perhaps because of the elevated research status of the UK Government, both Foresight and the report’s ‘Lead Expert Group’ (six white male professors at UK universities), Trapped Populations rapidly gained a solid foothold within the contemporary literature on environmental migration.^2^ Numerous scholars subsequently used the term to refer to people deemed geographically ‘trapped’ in environmentally high-risk areas due to economic, legal, or social constraints upon their mobility. Indeed, the rate at which ‘trapped’ populations were being identified soon after publication of the report suggests that some researchers readjusted the focus of their work to find evidence for involuntary immobility. Although such efforts highlighted the potential plight of individuals affected by involuntary immobility worldwide, this surge of interest in ‘trapped’ populations occurred from a foundation wrought with ambiguity.

Trapped Populations has already been problematised by Black et al. (2013) due to the conceptual difficulty of identifying affected people^3^ and the concept’s failure to adequately address a person’s ‘right to stay’ in a place that others may consider to be high-risk. The current academic definition of being ‘trapped’, proposed to be a possible (im)mobility outcome of the interactions between a person’s need and/or desire to migrate and their ability to do so (Black and Collyer 2014b), also does not lend itself well to the empirical methods common to migration research.^4^ However, launching into novel research without adequately accounting for the complex and multifaceted nature of immobility risks the imposition of externally formed ideals. To develop the concept in as cohesive and beneficial a manner as possible, we argue that it is important to explore both the roots of the concept and the way(s) it has been interpreted and applied in policy to date. A Critical Discourse Analysis (CDA) (e.g. Fairclough 1995, 2003) that focuses on the conceptual birth, development, and use of Trapped Populations is thus presented here to (1) understand why the concept appeared when it did; (2) explore how it has been shaped by environmental migration scholars to date; (3) identify the different way(s) the term is already being used; and (4) examine the potential for direct or inadvertent policy abuse/misuse of the concept in its current form.

## Discursive narratives and key literature on climate change-induced migration^5^

Although there are earlier references to the **environment as an important determinant of human mobility** (e.g. Wagner 1873; Durkheim 1899), during the twentieth century environmental explanations for displacement largely disappeared. It has been argued that this was a result of Western dichotomies that sought to separate nature and society (see Piguet 2013). At the hands of such division, scholars tended towards categorising the movement of people according to the various characteristics of the migrants, their motivations, origins, destinations, or duration of stay. From these characterisations, discursive narratives emerged that represented shared and accepted storylines seeking to explain migration, often in terms of binary opposites.^6^

Migration has now taken its place as a common term within the climate change discourse (Piguet 2013; Baldwin 2016), after a long debate around if environment, environmental change, and thus climate change potentially influence migration patterns (see for example Reuveny 2007; Hulme 2011). Despite current recognition (UNFCCC 2015, §50), a key narrative across much of the literature is the idea that **migration, displacement, and immobility due to climate change will occur in a distant future** (Baldwin et al. 2014; Baldwin 2016).

Widespread denial of the immediacy of climate change-induced migration perhaps explains the scarcity of research that has isolated the role of environmental stress as a sole determinant of migration decisions. Instead, the environment is often described as one of multiple contributing factors. This is clearly evident within the academic discourse where an ‘alarmist’ depiction (Dun and Gemenne 2008; Gill 2010) of a **growing number of ‘environmental refugees’** (Myers 1997; Bogardi and Warner 2009) has been supplanted by a more sceptical **common recognition that migration is driven by various factors**, of which climate change impacts may be one (Kibreab 1997; Castles 2003; Black et al. 2011a; Foresight 2011).

Efforts have long been made to **characterise the movement of people** according to the interactions imagined to be occurring between environmental stress, a person’s need/desire/willingness to be mobile, and the degree of control they can apply to their situation (e.g. Renaud et al. 2007; Black et al. 2013). Developing a better understanding of mobility decisions is important given the negative values that have been assigned to migrants and migration. Despite being critically analysed (e.g. Collyer 2006; McNamara 2007; Hartmann 2010) and problematised across disciplines (Said 1978, 1990; Anderson 1983; Bhabha 1994), narratives have emerged in recent years that frame **increasing migration flows as a security threat** (Weiner 1992; Smith 2007). Migrants are often described as a potential national security problem and emotively portrayed as an anonymous wave/tide/flood/stream of ‘Others’ moving across borders (Gill 2010; Piguet 2013).

A simultaneous discursive debate also took place in relation to the way environment-related moves were classified through terminology. The focus on ‘environmental refugees’ (e.g. El-Hinnawi 1985) that reached a crescendo in the 1990s was thus almost completely replaced by a **focus on ‘environmental migrants’**^7^ by the advent of the Foresight report in 2011 (Foresight 2011; Piguet 2013). When paired with continued interest in the situations of those people affected, this semantic adjustment from refugees to migrants might not seem overly important. However, a body of literature has emerged that sees it as a discursive move away from narratives around a conventional need for international protection and towards the reproduction of terminology based around ‘climate justice’ (McNamara 2007; Lister 2014). While the notion of an environmental refugee shed light on the ‘climate debt’ held by northern countries, some argue that such **moral obligations were lost, and economic or political agendas better disguised, in the move towards ‘environmental migrants’** (Hartmann 2010; Felli and Castree 2012; Hyndman 2012; Methmann and Oels 2015).

Instead of being referred to solely in terms of their potential status as victims in need of protection (Morrissey 2012; Hunter et al. 2015), people forced to move by environmental factors were simultaneously described as **environmental migrants with individual adaptive agency**. This allowed scholars to link the discipline with ‘limits to adaptation’, ‘climate resilience’, and ‘social transformations’ (e.g. Folke et al. 2002; Adger et al. 2009; Pelling 2010) and placed a stronger focus on individual ‘decision-making and behavioural studies’ (Lu 1999; Kniveton et al. 2011).

Foresight (2011) highlighted that migration could be considered a successful adaptation measure. However, the theoretical model proposed by the report has been accused of moving away from the collective socio-environmental context that may have contributed to environmental displacement and towards a mind-set that focuses upon an **individual’s capacity or ability, and thus indirectly an individualised responsibility, to adapt** (Felli and Castree 2012; Baldwin 2016). Expectations were thus proposed to have changed **from socio-political or socio-economic transformations towards encouraging individual resilience**. However, if each and every one of us is responsible for our own capacity to ‘bounce back’, it becomes difficult to unravel what happens to those people who are incapable, unfit, or for other reasons do not manage to adapt, migrate, or escape (Felli and Castree 2012; Baldwin 2016). The quiet supposition thus appears to be that ‘maladaptive’ migrants will be left behind and become ‘trapped’, having failed in their individual responsibility to be resilient.

Reproducing normative adaptive narratives and defining who is adapting successfully or being resilient by pursuing the ‘right’ climate action **may lead to affected people ending up less supported or more vulnerable** than before (Cannon and Müller-Mahn 2010; Eriksen et al. 2015; Ayeb-Karlsson et al. 2016). An individualised responsibility to adapt also implicitly assumes that even if all occupants of a locale do not share the same access to financial resources, they will share the same social, cultural, and emotional state and thus aspire towards the same behavioural response. This assumption becomes particularly problematic when confronted with the seemingly illogical immobility of people exposed to critical environmental threats. By not adapting or becoming resilient in the manner defined as correct by some external actor, affected people may thus become subject to interventions intended to facilitate their reintroduction into resilient mobility, a process described by some commentators as tantamount to promoting the circulation of cheap labour and maintaining existing hegemons (Felli and Castree 2012; Bettini 2014).

Given the apparently fragile nature of the Trapped Populations concept and its position within already contested literatures on environmental migration and migration as adaptation, this article seeks to further our understanding of the concept by critically analysing the different contexts in which the term has been used to date. If definitions of what constitutes a ‘trapped’ population are applied with too broad a brush, the rights of affected people could be threatened and existing inequalities and vulnerabilities further extended by placing the burden of adaptation on already fragile individuals.

## Theoretical framework and methodology

Text and language can be used to highlight changes in knowledge, attitudes, beliefs, and values. To understand how text works to shape our social realities, one needs to understand the relationships between human actors, structures (e.g. language), practices (e.g. order of discourse), and events (e.g. texts). As social agents, people have the power to influence societal structures and practices, and support the establishment of relations and value between elements of texts (Archer 2000; Fairclough 2003). Although their actions are not entirely socially determined, they are constrained by biases and opinions.

Because social interaction is undertaken through the production and distribution of spoken and written words, an effective means of understanding shared narratives is the analysis of discourse (Foucault 1981, 2002; Fairclough 2003). People position themselves within these ‘collectively shared domains of statements’ (see Foucault 1981) according to their identity and ‘world’ of social relationships. As a result, a discourse represents the perceived and interlinked realities that people position themselves within, not an objective reality. Discourses can complement or cooperate, compete, contradict, or dominate one another (Foucault 1981; Fairclough 2003).

The dual pairs generated by binary opposites (Said 1978, 1990; Foucault 2002) lock people into discourses and divert them away from important societal factors such as the power relations behind the dichotomy (Foucault 1981, 2002). Binary opposites thus define social groups in terms of both their members and non-members. Those ‘Others’ are assigned characteristics not wished upon the collective ‘we’ so that meaning and value are given to who they are (Said 1978, 1990; Foucault 1981, 2002; Bhabha 1994). Critical text analyses must therefore consider not only what dichotomies exist and how they are described, but also the ‘Habitus’, or ways in which the author, or the reality being described in the text, perceives and reacts to the social world around them (Bourdieu and Wacquant 1992; Fairclough 2003).

The analysis undertaken within this article seeks to detect different discourses around Trapped Populations that have emerged through the reproduction of different genres and styles to create shared realities, and critically acknowledges the power position of the relevant authors and their assumed ‘scientific hard-factual truth’ (Fairclough 1995, 2003). In this way, the CDA is not limited to the written words alone, but attention is drawn to the structure, meaning, and order of the described discourses.

Publications subjected to analysis were selected using online search tools *Web of Science, Google Scholar*, and the *CliMig* database (Piguet et al. 2017^8^) to identify those that used the word ‘trapped’ in the context of migration-related environmental immobility at least once. This focus restricted the selected publications to those released ‘post-Foresight’ with an explicit reference to Trapped Populations.^9^ Authors of such articles are proposed to have either consciously or subconsciously decided to reproduce the terminology that emerged from Foresight, a process of particular interest due to the powerful and influential scientific elite behind the report.

**Fig. 1:**
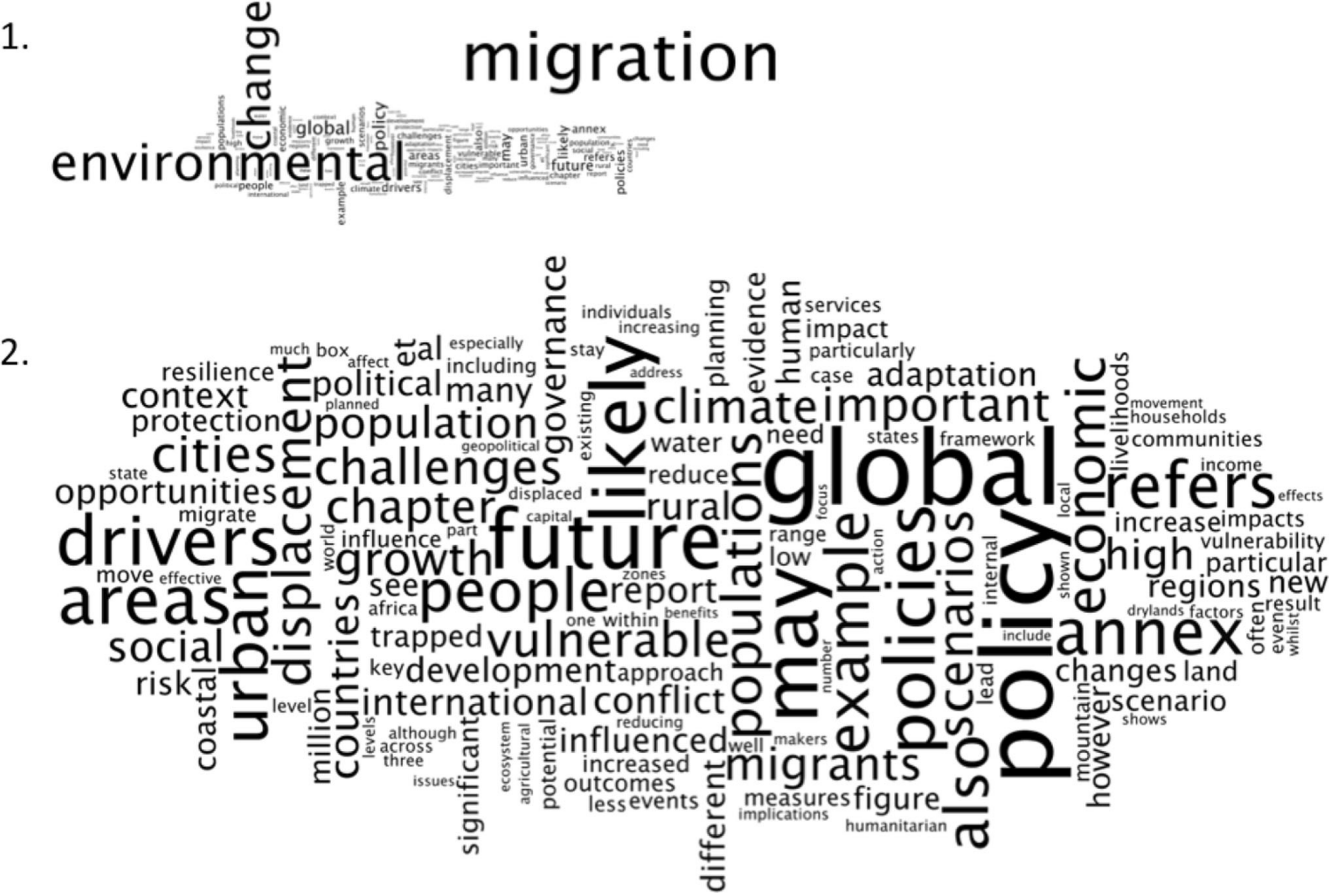
Word cloud 1 has been generated from the full text of the Foresight MGEC (2011) report and is dominated by the words *migration*, *environmental*, and *change*. To enable more in-depth analysis, Word cloud 2 has been created using the same source text but is displayed with the words *migration*, *environmental*, and *change* removed. Larger font size of a word indicates greater prevalence within the text with non-conceptual words such as ‘the’, ‘by’, and ‘for’ removed

Twenty-one academic texts (18 articles and three book chapters) were identified that met the search criteria. The frequency of occurrence of all words in each text was quantified using Wordle and associated ‘word clouds’ (Fig. 1) were used to highlight keywords and thus identify discursive narratives (as applied by Jorgensen 2015; Chambers 2016; Gardner 2017).^10^

To inter-discursively analyse each text (Fairclough 2003; Wodak 2011), careful attention was drawn to the following relationships:

- Semantic: Relations in meaning between expressions, words, sentences, and clauses over longer stretches of text such as reasons, consequences, and purposes, e.g. repeated descriptions and expressions of ‘trapped’ people as an *urgent problem* needing a *rapid solution*.
- Grammatical: Relations between morphemes, words, phrases, and sentences, e.g. references to ‘a’ trapped population or ‘the’ trapped population.
- Vocabulary: Patterns, re-occurrence, and co-occurrence between vocabulary, words, and expressions, e.g. trapped how? where? by what? or by whom?
- Phonological: Highlights or textual intonation through font style or size and the use of bold, italic, underlined, and quoted words, e.g. references to ‘trapped’ or “trapped”.

Each full text (minus references) selected for analysis was subjected to the following analytical procedure: (1) ‘word clouds’ were generated to gain an overview of key concepts, repetition of words, and discursive narratives; (2) text sections referring to ‘trapped’ were extracted for further analysis; (3) the discursive meaning and context describing Trapped Populations in each extract was analysed through the identification of semantic, grammatical, vocabulary, and phonological textual relationships (e.g. Fairclough 2003); (4) a short summary, including example extracts from the original text, was composed describing the discourse groups identified (see discourse group overview in Table 1).

**Table 1:** Overview of discourse groups

Authors	Year	Institutional affiliations during authorship	Type of publication	Cited Foresight report?	Author(s) linked to Foresight?	Number of times ‘trapped’ cited	Article keywords	‘Word cloud’ keywords
*Discourse A: Reproducing the Foresight narrative*								
Black, R. Bennett, S.R.G. Thomas, S.M. Beddington, J.R.	2011	UK university and UK Government	Peer reviewed journal	Yes	Yes, directly (all either members of Foresight or the Lead Expert Group)	Whole = 5 Abstract = NA Title = 0	NA	*migration, environmental, change, drivers, people, global, high, low, economic, migrate*
Adger, W.N. Arnell, N.W. Black, R. Dercon, S. Geddes, A. Thomas, D.S.G	2015	UK universities	Peer reviewed journal	Yes	Yes, directly (all members of the Lead Expert Group)	Whole = 6 Abstract = 2 Title = 0	migration, environmental change, adaptation, governance	*risk, climate, economic, social, global, populations, policy, conflict, areas, growth, international*
Adger, N. Adams, H.	2013	UK university	Book chapter	Yes—not in relation to being trapped	Yes (one member of the Lead Expert Group)	Whole = 1 Abstract = 0 Title = 0	NA	*environmental, migration, risk(s), change, mobility, vulnerability, resources, economic, people*
Black, R. Arnell, N.W. Adger, W.N. Thomas, D. Geddes, A.	2013	UK universities	Peer reviewed journal	Yes	Yes, directly (all members of the Lead Expert Group)	Whole = 13 Abstract = 1 Title = 0	environmental change, migration, extremes, mobility, displacement	*displacement, extreme, events, change, disasters, policy, vulnerability, change, economic, international, global*
Penning-Rowsell, E.C. Sultana, P. Thompson, P.M.	2013	UK university	Peer reviewed journal	Yes	Yes, indirectly (case study authors)	Whole = 6 Abstract = 0 Title = 0	hazards, Bangladesh, impacts, evacuation, migration, focus groups	*people, work, evacuation, migration, land, areas, hazards, affected, coastal, population*
Afifi, T. Milan, A. Etzold, B. Schraven, B. Rademacher-Schulz, C. Sakdapolrak, P. Reiff, A. van der Geest, K. Warner, K.	2015	Consortium of German research organisations, primarily UNU-EHS	Peer reviewed journal	Yes—not in relation to being trapped	No	Whole = 4 Abstract = 1 Title = 0	human mobility, rainfall variability, food security, climatic stressors	*migration, households, rainfall, research, food, variability, adaptation, human, livelihoods, migrants, insecurity, case, study*
Gray, C. Wise, E.	2016	US university	Peer reviewed journal	No	No	Whole = 2 Abstract = 0 Title = 0	NA	*climate, migration, effects, migrants, data, specification, households, results*
Milan, A. Ruano, S.	2014	UNU-EHS and Guatemalan Ministry	Peer reviewed journal	Yes	No	Whole = 5 Abstract = 1 Title = 0	climate variability, migration, Guatemala, food security, mountain	*food, migration, households, research, climate, change, production, variability, local, data, patterns, survey*
Warner, K. Afifi, T.	2014	UNU-EHS	Peer reviewed journal	Yes - not in relation to being trapped	No	Whole = 9 Abstract = 0 Title = 0	adaptation, climate change, vulnerability, migration	*migration, hh(s)* (abbreviation of household(s))*, rainfall, food, research, livelihood(s), variability, security*
*Discourse B: Expanding the Foresight report*								
Geddes, A. Adger, W.N. Arnell, N.W. Black, R. Thomas, D.S.G.	2012	UK universities	Peer reviewed journal	Yes - not in relation to being trapped	Yes, directly (all members of the Lead Expert Group)	Whole = 12 Abstract = 1 Title = 0	international migration, internal migration, environmental change, governance, security, displacement, protection	*migration, environmental, change, governance, states, international, people, move, displacement, economic*
Geddes, A.	2015	UK university	Peer reviewed journal	Yes	Yes, directly (member of the Lead Expert Group)	Whole = 11 Abstract = 1 Title = 0	migration, climate change, security South Mediterranean	*migration, environmental, change, effects, people, governance, climate, security*
Humble, A.T.	2014	Earth League, Sweden	Journal article	No	No	Whole = 8 Abstract = 1 Title = 1	NA	*border(s), migrants, trapped, security, access, countries, migration, dangerous, protection, populations, areas, forced, safer*
Sow, P. Marmer, E. Scheffran, J.	2015	German universities	Peer reviewed journal	Yes - not in relation to being trapped	No	Whole = 1 Abstract = 1 Title = 0	West African migration, climate change, Morocco, racism	*migrant(s), migration, climate, country(ies), development, change, social, destination, origin, immigrants*
Black, R. Collyer, M.	2014 (book)	UK universities	Book chapter	Yes	Yes (one member of the Lead Expert Group)	Whole = 68 Abstract = na Title = 1	NA	trapped, populations, move, migration, may, policy, need, research, individuals, immobility, ability, crisis, movement, humanitarian *
Black, R. Collyer, M.	2014 (FMR)	UK universities	Journal article	Yes	Yes (one member of the Lead Expert Group)	Whole = 22 Abstract = 1 Title = 1	NA	*trapped, move, mobility, migration, populations, policy(ies), crisis, access, resources, ability, unable, movement*
Bhatta, G.D. Aggarwal, P.K. Poudel, S. Belgrave, D.A.	2015	Global research partnership (CGIAR) members (Canada and India) and Canadian city.	Peer reviewed journal	No	No	Whole = 7 Abstract = 1 Title = 0	distress migration, climatic risks, extreme events, rainfall variability, gender dimensions, South Asia	*migration, household(s), women, events, adverse, farmers, income, climate(ic), development, community*
Adams, H.	2016	UK university	Peer reviewed journal	Yes	No	Whole = 22 Abstract = 3 Title = 0	immobility, trapped, place attachment, behavioural theory, environmental change, migration, Peru	*migration, change, place, mobility, environmental, population(s), location, people, climate, attachment, dissatisfaction, negative, trapped*
Hillman, F. Ziegelmayer, U.	2016	German research institutes	Peer reviewed journal	Yes - not in relation to being trapped	No	Whole = 6 Abstract = 1 Title = 0	migration, environmental change, climate change, migration trajectories, Ghana, Indonesia	*migration, environmental, change, population, trajectories, migrant(s), out*-*migration*
*Discourse C: Opposing the Foresight report*								
Baldwin, A.	2016	UK university	Peer reviewed journal	Yes	No	Whole = 1 Abstract = 0 Title = 0	climate change, migration, race, neoliberalism, affect	*migration, climate, change, affect(ive), white, racial, discourse, future, race, neoliberalism, state, biopower*
Baldwin, A. Gemenne, F.	2013	UK and French universities	Book chapter	Yes - not in relation to being trapped	No	Whole = 2 Abstract = 0 Title = 0	NA	*migration, climate, change, research, empirical, change*-*induced, social, policy, political, environmental, refugees*
Felli, R. Castree, N.	2012	UK university	Peer reviewed journal	Yes	No	Whole = 2 Abstract = 0 Title = 0	NA	*environmental, migration, report, social, adaptation, change, global, neoliberal, state, individual, policy*

*NA* no article keywords were specified for a particular publication

* A situation where full digital text was not available for use in the creation of a ‘word cloud’. In such an instance, a ‘word cloud’ was created from the excerpts taken from the article due to their relation to Trapped Populations

## Analysis: The Foresight report

To create a baseline for comparison, our analysis begins with the discourses presented in the Foresight report. Inter-discursive analysis of the semantic relationships found in the text reveals three clear narratives, summarised below.^11^

### Discursive narrative 1: Climate change, threats, and challenges are on the way

The first narrative feeds into the climate-changed future perspective (Baldwin 2016) outlined previously and describes the notion that a situation of **threats** and **challenges** will emerge in the near **future**. The recurrence of expressions such as **decades ahead** and **future threats**, and the use of future tense places the problem ahead of us.

**Extract 1** 12

***The impact of environmental change on migration will increase in the future***. In particular, environmental change may **threaten** people’s livelihoods, and a traditional response is to migrate. **Environmental change will** also **alter populations’ exposure to natural hazards**, and **migration is,** in many cases, **the** ***only*** **response** to this. For example, **17 million people were displaced by natural hazards in 2009** and **42 million in 2010** (this number also includes those displaced by geophysical events).

(Foresight 2011:9)

The **challenges** described are proposed to include **population** movements, and **cities grow(ing) in size** due to **new urban migrants** or **rural–urban migration** and refer to **millions of people** being affected. The picture painted is much in line with the Peace and Security narrative (e.g. Said 1978; Barnett 2003), where a moving or stagnated mass of people is considered a security **threat**. However, instead of being presented as a threat to national security, the **challenge** is described as a global problem that merits a global solution by its nature as a **concern for the international community**. The binary opposites that define ‘us’ and ‘them’ thus expand beyond the national scale to identify a shared consensus that, for example, **cities in low-income countries are a particular concern**. ‘The Others’ identified by the report are thus expected to originate in impoverished locations where the disorder is anticipated to start.

### Discursive narrative 2: Global well-managed policy planning is the solution for safety

The second narrative furthers the depiction of an imagined global ‘we’ by mention of **the international community** for whom ‘trapped’ populations represent an important **policy concern**. The report describes **planned** and **well-managed** migration as the **action** this ‘community’ should pursue but includes no critical reflection with regard to who is a part of this group and who is not.

The binary opposites are clear with the disorder, **challenge**, **concern**, or **threat** on one side that must be **managed**, **planned for**, **reduced**, and **avoided** by the other. Order and safety is achievable through **proactive** and **well-managed** policy planning. The solution is not to **prevent** migration but to **facilitate**, **plan**, and **manage** its occurrence. People becoming displaced or **trapped in vulnerable rural areas** would lead to **graver outcomes** or **raise wider challenges**. The **rural–urban** relationship portrayed by Foresight offers another binary opposite and occupies a central role in the report’s descriptions. People are referred to as **trapped in vulnerable rural areas** with managed migration to urban areas presented as a possible solution. Although the report acknowledges that people may end up trapped in cities, this concern is placed alongside climate change as occurring in the future.

One reading of this narrative (whether intentional or not) is that it reproduces Western dichotomies where rural places are considered vulnerable and primitive problem areas, with urban areas portrayed as modern and holding the keys to success. This portrayal possibly relates back to the authorship of the report or at least the repetition of a western narrative around place, space, and culture. The narration of a safe, managed, and successful rural–urban migration locates the solution in an urban context and acknowledges the migrant as a potential adaptive agent. A picture is thus painted of an individual **building resilience and transforming adaptive capacity**, a situation that separates them from a homogenous moving mass.

### Discursive narrative 3: To stay safe economic progress and resource protection

The third narrative identified links a proactive response to the achievement of longer-term gains. The report describes vulnerability in economic terms as a **lack of capital** and **wealth** so that poor people are trapped in **low-income countries**. Their **reduced level of capital** makes them **unable to move away** from environmental threats in a simple linear fashion. People cannot therefore end up trapped so long as they are able to buy a bus ticket to a new location. However, the assumed simplicity of this linear economic relationship ignores the potential for social and psychological factors to trap people in dangerous locations alongside, or instead of, financial constraints.

Despite the report’s promotion of the financial benefits of managed migration, a critical perspective raises the possibility of another side of the story. Indeed, a proactive approach to managing migration may serve to **capitalise** upon and **maximise** the **benefits** of migration for other, larger-scale actors. As a result, the narrative also warns of the dangers of not applying the sort of proactive policy solutions described. This critique feeds into the binary nature of disorder and order. If *we*, **the international community**, do not apply the **proactive policy** approach of **planning** and **managing** the migration flows, *we* will be facing a world filled with **conflict** and **tension** over **natural resources**. The report emphasises that the **relationship between poverty, resources, and conflict** will trap **poor** people into **conflict situations** unless there are international **policies to avoid** and **address** this issue.

## Analysis: Selected publications

At the time of this review, 21 publications (18 articles and three book chapters) had been published post-Foresight containing the word ‘trapped’. Four of the publications have single authors with nine, including the three book chapters, having dual authorships. The complete set of contributors stands at 40 authors, of whom 11 are women. 33 authors belong to European institutes, including 17 in the UK and 14 in Germany. Because a concept must be described numerous times to become discursively repetitive, those texts with more references to ‘trapped’ were subjected to greater discursive scrutiny. Of the total, 14 articles and one book chapter refer to the ‘trapped’ more than three times (Table 1).

Using the Foresight report as a comparative baseline, three discourse groups were identified: publications reproducing the Foresight narrative (Discourse A); publications reproducing *and* expanding the Foresight narrative (Discourse B); and publications opposing the Foresight narrative (Discourse C). Additionally, within discourse groups A and B notable differences are evident between publications authored by the Foresight Lead Expert Group and those by scholars who are not Foresight report authors.

### Discourse A: Reproducing the Foresight report

Discourse A consists of nine publications reproducing the narrative conveyed within the Foresight report. Four articles (Black et al. 2011b, 2013; Adger and Adams 2013; Adger et al. 2015) include at least one Lead Expert Group author, one is by authors linked to a Foresight-commissioned case study (Penning-Rowsell et al. 2013) and four did not include Foresight authors (Milan and Ruano 2014; Warner and Afifi 2014; Afifi et al. 2015; Gray and Wise 2016).

The publications contributing to Discourse A by reproducing the Foresight narrative do so in different ways. Three articles authored solely by Lead Expert Group members (Black et al. 2011b, 2013; Adger et al. 2015) refer to ‘trapped’ in a manner much in line with the both the original Foresight description and the three discursive narratives identified above. **Populations** are thus portrayed as a future **critical risk** needing to be solved by supporting people to **migrate**, an action representing a **well-documented** way to **effective(ly) adapt**.

Questions of why people will become ‘trapped’ are strongly narrated around economic language where **immobility** is **cause(d)** by **people losing their assets**, **falling into poverty traps**, or suffering from **a lack of capital**. Although differences between **financial**, **social**, and **human capitals** are acknowledged, the discursive relationship between capital and immobility is strongly **economic** and focused on **financial capital**. For example, the narrated relationship of **fear** around immobility is framed in terms of a **fear of what would happen to property or assets left behind**. **Vulnerability** is also linked to **wealth** so that **trapped populations** are seen as being **vulnerable without the ability or resources to move.**^13^

Most of the additional articles belonging to Discourse A are case study based. When referring to who is ‘trapped’, **households** and **communities** rather than individuals are identified. Little in the way of critical reflection is found on who is a part of the household/community, or whether the entire unit of people are ‘trapped’. The **vulnerability** and **immobility** described are strongly **economic(ally)** determinant but also focus on **livelihood**, **income, assets**, and **food security**. This shift in language links back to the **worse-off household** Foresight narrative. Whole **HHs** or **communities** are thus described as being at **risk of becoming** or are **trapped due to lack of resources, assets** and **means**, **extreme poverty**, or **substantial economic losses**.

The nature of economic losses and their link to climate change are described by all articles in this discourse group in the same terms as the Foresight report. It is an event that will occur in a **near future**. Throughout their conceptual reproduction, this group makes frequent references to the **Foresight report** and articles by Foresight lead authors (e.g. Black et al. and Adger et al.). Trapped Populations and references to involuntary immobility consistently appear in quotation marks (e.g. **“trapped”**, **“immobile”**, and **“trapped populations”**).^14^

### Discourse B: Expanding the Foresight report

Consisting of a further nine publications, Discourse B both reaffirms the Foresight narrative and offers some expansion of the concept. Four of the nine publications have at least one Foresight Lead Expert Group author (Geddes et al. 2012; Black and Collyer 2014a, b; Geddes 2015), while five publications did not include Foresight authors (Humble 2014; Adams 2016; Bhatta et al. 2015; Hillmann and Ziegelmayer 2016; Sow et al. 2015). Although the publications do feed into the Foresight narratives, two clear expansions have been identified:

#### Expansion of the future threats and challenges—even darker and more urgent

The Foresight narrative around a climate-changed future of challenges is built upon to provide more details of what darkness lies ahead. The challenges and threats described in Foresight Discursive Narrative 1 are intensified throughout the publications (Geddes et al. 2012; Humble 2014; Geddes 2015; Sow et al. 2015).

The subjects portrayed as being at risk of becoming ‘trapped’ are described as the **people**, **migrants**, and **immigrants** that constitute the **tens of millions of people** or **growing number of people** that are expected to pose **a governance challenge** to nation **states**. Binary opposites such as we and them, **conflict** and **protection**, or **danger** and **security** are strongly reproduced. An alarmist rhetoric also describes **hostile situations** where **migrants are trapped on the ‘wrong side’ of the border unable to access legal protection or basic social necessities**.

The linguistic reproductions used within Discourse B present some changes in the use of Trapped Populations: (1) instead of being rendered immobile in environmental high-risk areas, people are described as trapped within **states**, e.g. **trapped in their own countries or in transit countries** and **due to border security**; (2) people are narrated as **trapped in situations** rather than geographic areas; (3) instead of lacking economic resources, focus is on affected peoples’ lack of **legal protection frameworks**; and (4) the role of environmental change has been reduced so that those ‘trapped’ include people **displaced** due to **conflicts** and **economic migrants moving towards large(r),** richer **cities** and **states**, such as **towards the EU**.

In addition to the Foresight narrative on future challenges, Discourse B also feeds into narratives describing **increasing migration flows as a security threat** and debates on **refugee or migrant protection**. It is, for example, stated that **migrants’ circumstances fall within legal protection frameworks** but they are **trapped on the ‘wrong side’** of these frameworks. People thus face **dangers** to the extent of **discrimination**, **racism**, **hostility**, **violence**, **physical and sexual abuse, forced labour, human trafficking, and organ theft.**^15^

#### Expansion of the economic reasoning—it is more complex

The second expansion beyond the Foresight narrative comes from five publications (Black and Collyer 2014a, b; Adams 2016; Bhatta et al. 2015; Hillmann and Ziegelmayer 2016), two of which (Black and Collyer 2014a, b) involved a Lead Expert Group author. There are overlaps with the previous discursive expansion through the way ‘trapped’ includes legal situations as well as locations in which people may become ‘trapped’.

These publications share the idea that Trapped Populations had not been adequately problematised, with the reality being more **complex** than originally portrayed by Foresight. As a result of this complexity, the authors propose an expansion of the concept to accommodate different perspectives. These include the relevance of social and legal access in relation to, for example, gender as well as fear and emotional impacts upon decision-making in relation to place attachment.

Black and Collyer’s (2014a, b) publications differ greatly in length but overlap in message.^16^ They serve as expansion initiators towards the acknowledgement of a greater degree of complexity in a number of ways by (1) referring to **individuals** (as well as people and populations) and thus recognising that whole units of people do not necessarily end up trapped; (2) referring to people ‘trapped’ in **situations** and **conditions** as well as geographic areas; (3) acknowledging, but also criticising, the economic **resource** focus of Trapped Populations and expanding the **multifaceted** reasoning to include access to **social networks**, **marginalisation**, and social stigmas as important factors; (4) emphasising that individuals may end up trapped at **any stage in their migration process**, thereby being **partially mobile yet trapped,** especially in **refugee situations**; and (5) referring to the conceptual necessity for both a ‘**want** and **need**’ to move, as well as including consideration of those **offered** an **opportunity** to move but who **refuse to leave.**

At the hands of this complexity, a strong narrative emerges around the **limited information, research**, and **understanding** of the concept. Black and Collyer recognise the valuable insights of the **Foresight report** and do not oppose its storyline but build upon the foundations laid at the conception. However, although **policy** was described by Foresight as a potential solution to the ‘problem’, Black and Collyer encourage more caution of policy measures until our understanding of the concept, through more and better research, has increased.^17^

The remaining publications contributing to Discourse B (Adams 2016; Bhatta et al. 2015; Hillmann and Ziegelmayer 2016) share Black and Collyer’s aim of expanding upon the complex and multifaceted nature of Trapped Populations. Hillmann and Ziegelmayer refer heavily to Black and Collyer’s (2014a) conceptual contributions and cite **claims** around the existence of **“trapped populations”**. Bhatta et al. seek to expand our understanding of **social, cultural, religious**, and **emotional restraining** elements on mobility in relation to women, children, and elderly. The expansions are made not only in relation to *why* people get ‘trapped’ but also in terms of *who* ends up ‘trapped’. The article refers to **trapped group(s)** synchronising demographically ‘trapping’ elements such as **gender** and age. The concept is described in terms of **dynamic vicious cycles where women and their children get trapped.**^18^

Emotional attachment to place is mentioned by Bhatta et al. and Hillmann and Ziegelmayer. Adams, however, places greater focus on this aspect to **expand** the **view** of **what it means to be ‘trapped’ through insights from social and behavioural theories**, using **residential**, **place attachment**, and **social capital** to explain why **rural populations across the globe decide to remain** in a location **despite dissatisfaction**. In so doing, Adams seeks to contrast the **traditional** or **current definition** of Trapped Populations by (1) focusing on **individuals** instead of **households**, **people**, and **populations**; (2) acknowledging the **subjective dimensions** and **differentiated capacity** to which a **“single” population respond** and **experience impacts**; and (3) expanding the notion of ‘trapped’ to include *situations* where people **are physically unable to leave**, **without the** financial **resources** or **means to escape.**^19^

Authors contributing to the second avenue of expansion identified within Discourse B argue to some extent for the complex nature of Trapped Populations and the need for further research to bolster academic insight. Agreement is broadly reached on the limited value of a purely economic assessment of involuntary immobility, but the consistency with which that narrative is adhered to across the five publications is limited. Despite efforts to expand upon the **traditional** definition initiated by Foresight, respondents contributing to findings refer to **lack of money**, **property**, and **house** as the key factors in their immobility.^20^

In order to move beyond an arena where caution can be replaced by confident and effective policies, research tailored to accommodate the unique and complex nature of the concept will be necessary. In this way, some of the publications appear to have ended up ‘trapped’, or on the move between the two discourse groups. The texts are reproducing elements of Discourse B but also, at times, falling back into narratives of Discourse A.

### Discourse C: Opposing the Foresight report

Discourse C consists of three publications by external authors opposing the Foresight narratives (Felli and Castree 2012; Baldwin and Gemenne 2013; Baldwin 2016). These texts problematise Trapped Populations and highlight the dangers of labelling people as ‘trapped’. Discourse C thus competes with Discourse A and, in some ways, with Discourse B. In contrast with the other discourse groups, the publications contributing to Discourse C do not heavily repeat the word ‘trapped’. The word ‘Foresight’ is, however, repeated 26 times across the three texts. Discourse C authors are thus critiquing Trapped Populations as a single aspect of the Foresight report’s wider findings.

Felli and Castree (2012) offered instant opposition to the release of the Foresight report by highlighting the dangers of **promoting migration as adaptation**. The authors oppose the third Foresight narrative that promotes well-**managed** and planned **global migration policies** by suggesting that the notion of a **trapped population** may be used to **justify** the promotion of **a new global reserve army of labour** while appearing to be **advocating policy of open borders**. Felli and Castree’s perspective proposes that the Foresight promotion of migration that will create economic and **developmental benefits** for **migrants**, **countries of destination**, and migrant **states** or **territories** through **remittances** is flawed. The concept is thus described as a means to **justify** the **uncritical promotion of “temporary and circular migration schemes”** that **allow** ‘trapped’ people to **escape suffering** in **environmentally** dangerous **areas** without clearly stating the wider economic gains occurring as a result. Elements of this criticism of the Foresight global policy solution and the associated risks around **neoliberalism** are also raised by Baldwin and Gemenne (2013).^21^

Baldwin (2016) presents a more in-depth analysis of the Foresight report and expands the warning raised by Felli and Castree by linking the descriptions used by Foresight to power, discourse, and race. The article highlights the dangers of **maximising adaptive migration in the interest of capital circulation** and warns against the installation of an **affective infrastructure** that **obscures** and conceals **racial management** and defines or **stipulates maladaptive migration**.^22^ These cautions align well with some of the discursive narratives detected in our literature review. These include post-colonial descriptions of environmental mobility through the ‘new language of climate change’, the dangers of an individualised responsibility to adapt, and the risks associated with defining someone as either an adaptive/resilient or maladaptive/non-resilient migrant. While the successfully adaptive migrant remains mobile and productive, a maladaptive migrant becomes ‘trapped’. Even when climate action or adaptation support programmes are constructed to protect people, labelling them as ‘trapped’ has the potential to do more harm than good; people may end up even more vulnerable, less supported than before, or having their rights violated.

## Discussion

It is interesting that one of the key findings of a well-funded UK Government report produced by migration experts commissioned to investigate how people will move in the future due to climate change impacts highlighted non-migration as a potential threat. Although this danger was framed by the Foresight Lead Expert Group in terms of its humanitarian consequences, the threat posed by such immobility to the existing status quo must also be considered. The power effects of language, vocabulary, and meaning are of particular importance within policy. For example, the inclusion of ‘*displacement, migration and planned relocation in regards to climate change*’ through §14f in the 2010 UNFCCC Cancun Agreements marked a unique linguistic breaking point in how migration was framed in relation to climate change (UNFCCC 2011, §14f). Resettlement suddenly entered the rhetoric on how to protect vulnerable populations from the future threats of climate change (e.g. Dun 2011; Stal 2011; Iftekhar and Darryn 2014). However, the critical perspective presented within Discourse C suggests that any policy interventions intended to prevent or aid ‘trapped’ individuals must tread carefully when dealing with uncertainties inherent to future environmental changes. Seemingly noble intentions must not be rolled out without adequate consideration of their wider consequences.

The CDA presented here was used to shed light on how and why certain narratives and realities surrounding Trapped Populations were shaped in specific ways. The analysis revealed a clear conceptual storyline emerging from the Foresight report. Three discourse groups were identified that continued the story. However, discourses do not exist in isolation and the original Foresight narrative has been shown to have dominated Discourse A (reproducing), complemented and cooperated with Discourse B (reproducing and expanding), and been contradicted and competed with by Discourse C (opposing). A deeper critical analysis of the language reproduced through the Foresight report, such as the strong economic and possibly post-colonial descriptions, might be traced back to the commissioning of the report. As an aside, it is worth comparing the language and authorship in promotional videos of the Nansen Initiative and the Foresight report.^23^

Similarly, the suggested solution of planned and controlled migration, resettlement, or relocation programmes must be examined in the light of governance and its power effects. Migration scholars have warned against the assumption that mobility is the panacea needed (Hartmann 2010; Black and Collyer 2014b). Nonetheless, frequent mentions of planned and well-managed migration within the Foresight report and Discourses A and B suggest that proactive assistance measures such as assisted migration, relocation, or resettlement may be promoted as an effective and favourable climate action solution for ‘trapped’ populations. However, the ideology behind such proactive forms of policy recommendations or ‘assistance’ requires careful management to ensure that they preserve the autonomy of affected people.

In situations where immobility is involuntary and people willingly self-identify as ‘trapped’, assisted migration similar to that initiated when a refugee is offered ‘refuge’ in a safe state may be welcomed. However, where immobility is voluntary, it will represent an imposition into the lives of people who do not want to leave their homes (Hess et al. 2008; Adger et al. 2011). Climate policy recommending resettlement and relocation must be approached in a manner that reflects the incredibly complex and sensitive nature of the process (Hansen and Oliver-Smith 1982; de Sherbinin et al. 2011) and acknowledges the power and prejudices that may underlie its use.

## Conclusion

Although Trapped Populations has been described and interpreted in a number of ways, the concept is still developing and differences continue to emerge in the ways that it is defined. This article has sought to make a crucial contribution to the literature on the concept by drawing together all relevant post-Foresight references and offering an analytical template from which to create a cohesive understanding of the current state of the art. The CDA approach used has revealed that narratives around Trapped Populations have, to date, centred on the possibility of people becoming involuntarily immobile in dangerous locations in the future. However, the three main discourses identified across the 21 publications suggest that the concept has not developed in a clear and consistent way. After a fast and straightforward birth, the troubled teenage years of Trapped Populations look set to continue with some years on the backpacking trail to look forward to before it either fades into insignificance or strides forth in a more mature and stable form. The current fragmented nature of the concept and its irreducible nature in practical and theoretical terms has hindered its effective development and instead created a potentially dangerous policy tool. In its current form, there is a risk that the concept may be misused to seemingly ‘protect, save or move vulnerable populations from risky places’ while ensuring political or economic gain.

The theoretical and methodological approach used in this research is intended to remind us that language, texts, ideas, concepts, and knowledge are flexible, elastic, and constantly changing according to social structures. The power contained within language, and the way narratives turn into storylines, discourses, and reality should not be overlooked, especially not in relation to the risks, aftereffects, and dangers of describing someone as ‘trapped’. Referring to a person as ‘sick’ may lead to them being perceived as fragile, worthy of pity, or infectious and thus treated differently by other people. In the same way, labelling a person as ‘trapped’ has the potential to reduce or remove an individual’s agency, autonomy, and independence in determining their own destiny.

The human penchant for binary opposites should perhaps have helped us to envisage that after decades of alarmist warnings that “here comes the flood”, cautionary tales of the danger of standing waters would follow. Regardless, given the complex origins and multidisciplinary nature of Trapped Populations it is important that future progress around the concept, including how it is to be implemented through climate policy recommendations, is undertaken in a manner that recognises the linguistic power of the term and the potential ramifications of its use. In order to better understand migration flows and preserve the rights of affected people, greater effort must be made to dissect migration decisions and (im)mobility.

## Electronic supplementary material

Below is the link to the electronic supplementary material.

Supplementary material 1 (PDF 184 kb)

## Footnotes

1 Trapped terminology: In this article, Trapped Populations is used to refer to the existence of the concept (noun); ‘trapped’ is used to refer to when a person is labelled as being thus (adjective); and trapped is used to refer to the action of being rendered immobile in such a way (verb).

2 The Foresight MGEC initiative was a major pioneering and comprehensive review of most empirical evidence at the time on the linkage between environmental change and migration. The unique involvement of top-ranked universities and well-known research institutes, all in all about 350 experts based in over 30 countries representing a diverse set of disciplines, was influential in giving the report its elevated status. The two main aims of the report, to (1) develop a vision for how future population movements until 2060 would be influenced by global environmental changes and to (2) identify and consider the choices and decisions that policy-makers needed to take to create resilient climate policies in an uncertain future (Foresight 2011, p. 10), also in a way provided national governments and world leaders with a handbook on how to best ‘manage and control’ migration flows.

3 Also noted by Black et al. (2013, S36), “*the notion of a ‘trapped’ population is not a straightforward one, in scientific terms, not least because it is as difficult to distinguish, either conceptually or in practice, between those who stay where they are because they choose to, and those whose immobility is in some way involuntary*”.

4 It is, for example, difficult to capture someone’s subjective feelings, emotions, and nuances around desire, aspiration and ability to move in a survey questionnaire or a focus group discussion.

5 Bold font is used in this section of the article to highlight those narratives that are being identified as having become discursive. This article refers to ‘discursive narrative(s)’ to highlight the ways that text and language create and reproduce discourses, as well as destroying and challenging them. A narrative or story becomes discursive when it is repeated or reproduced enough to becoming a norm, socially shared attitude, truth, or reality by people engaging in the discourse.

6 The term ‘binary opposites’ in this article refers to a situation where a pair of related terms that are opposite in meaning are defined against one and another. The system was seen as a fundamental organiser of all languages and thoughts by structuralists such as Ferdinand de Saussure and Michel Foucault, e.g. man–woman, body–soul, black–white, east–west, and rural–urban.

7 First suggested in the 1970s (e.g. Brown et al. 1976), the term ‘environmental refugee’ was the focus of much debate before being largely discredited on the basis of both the legal definition (UNHCR 1967) and the perceived multi-causal nature of migration (Foresight 2011). The definition of ‘refugee’, provided under the 1951 UN Convention relating to the status of Refugees Article 1A and amended by the 1967 protocol, requires a person to be fleeing a fear of persecution or violence, neither of which can be legally defined as occurring at the hands of the environment. In recent years, narratives criticising the reluctant use of ‘climate refugee’ have appeared. Particularly, in relation to certain geographic areas, such as the disappearing islands in the Pacific, and the melting glaciers in the Arctic (e.g. Maldonado et al. 2013; Bronen 2014; Dreher and Voyer 2015; Kelman 2015).

8 The CliMig database can be found at the following address: https://www.unine.ch/geographie/climig_database (last accessed 01.12.2017). For more information on the database see (http://www.environmentalmigration.iom.int/projects/climig).

9 Those publications that referred to ‘trapped’ only in the bibliography did not meet the selection criteria. To maintain a focus on the linguistic development, changes of meanings, values, and narratives around Trapped Populations, publications containing only descriptive synonyms of being ‘trapped’ (e.g. ‘environmental immobility’ or ‘climatic involuntary immobility’) were not included in the analysis. The post-Foresight sampling used by this study was corroborated by the fact that repeated searches for appropriate references to being ‘trapped’ in academic publications from before 2011 showed no results referring to the concept. The report itself also does not contain any references to other publications that refer to the concept. The authors acknowledge that this selection narrows the analysis and excludes a wide corpus of literature referring to ‘involuntary immobility’ prior to Foresight, such as the research that followed Hurricane Katrina and Rita in the US (e.g. Elliott and Pais 2006; Stein et al. 2010; Thiede and Brown 2013, etc.). However, the decision to limit the analysis to literature referring to ‘trapped’ only is well in line with the selected methodology. Similarly, discourse analysis carried out on, for example, a specific debate/topic in mass media/policy (e.g. Gale 2004; Carvalho 2005; Boykoff and Boykoff 2007; KhosraviNik 2010) is better limited to articles published during this specific moment/area. This article does not aim to empirically investigate immobility per se, but to critically review the linguistic appearance and use of the concept Trapped Populations. The conceptual time of creation is important, since appearances of ‘new’ words and expressions in language generally take place for specific political, social, or cultural reasons.

10 Wordle is an online ‘word cloud’ generator available at www.wordle.net. The tool is useful when carrying out a textual discourse analysis as the clouds are accompanied by a count list of repetitions of words in the specific text being analysed (as applied in Jorgensen 2015; Chambers 2016; Gardner 2017).

11 Based on keywords from extracts 1 to 11.

12 All formatting represents the original publication appearance except bold text that has been applied by the authors to draw attention to keywords supporting the inter-discursive textual relationship. In the case where bold was used in the original text it has been turned into italic. Extract 1 is included in the main text as an example of the original manuscript text referencing system used by this article. Extracts 2 to 55 are appended to this document as supplementary information.

13 Based on keywords from extracts 12 to 14.

14 Based on keywords from extracts 15 to 21.

15 Based on keywords from extracts 22 to 29.

16 See extracts 30–31 for an example.

17 Based on keywords from extracts 30 to 38.

18 Based on keywords from extracts 39 to 44.

19 Based on keywords from extracts 45 to 49.

20 Based on keywords from extracts 50 to 51.

21 Based on keywords from extracts 52 to 54.

22 Based on keywords from extract 55.

23 See https://www.youtube.com/watch?v=D_A4l0qwF4g for the Nansen Initiative video, and http://www.bis.gov.uk/foresight/our-work/projects/published-projects/global-migration for the Foresight report launch video.

## References

[CR1] Adams H (2016). Why populations persist: Mobility, place attachment and climate change. Population and Environment.

[CR2] Adger, W.N., and H. Adams. 2013. *Migration as an adaptation strategy to environmental change*. World Social Science Report Changing Global Environments, 261–264. Paris: OECD Publishing/UNESCO Publishing.

[CR3] Adger WN, Dessai S, Goulden M, Hulme M, Lorenzoni I, Nelson DR, Naess LO, Wolf J (2009). Are there social limits to adaptation to climate change?. Climatic Change.

[CR4] Adger WN, Barnett J, Chapin Iii FS, Ellemor H (2011). This must be the place: Underrepresentation of identity and meaning in climate change decision-making. Global Environmental Politics.

[CR5] Adger WN, Arnell NW, Black R, Dercon S, Geddes A, Thomas DSG (2015). Focus on environmental risks and migration: Causes and consequences. Environmental Research Letters.

[CR6] Afifi T, Milan A, Etzold B, Schraven B, Rademacher-Schulz C, Sakdapolrak P, Reif A, van der Geest K, Warner K (2015). Human mobility in response to rainfall variability: Opportunities for migration as a successful adaptation strategy in eight case studies. Migration and Development.

[CR7] Anderson B (1983). Imagined communities: reflections on the origin and spread of nationalism.

[CR8] Archer MS (2000). Being human: The problem of agency.

[CR103] Ayeb-Karlsson S, van der Geest A, Ahmed I, Huq S, Warner K (2016). A people-centred perspective on climate change, environmental stress, and livelihood resilience in Bangladesh. Sustainability Science.

[CR9] Baldwin, A., and F. Gemenne. 2013. The paradoxes of climate change and migration. World Social Science Report Changing Global Environments, 265–268. Paris: OECD Publishing/UNESCO Publishing.

[CR10] Baldwin A (2016). Premediation and white affect: climate change and migration in critical perspective. Transactions of the Institute of British Geographers.

[CR11] Baldwin A, Methmann C, Rothe D (2014). Securitizing ‘climate refugees’: The futurology of climate-induced migration. Critical Studies on Security.

[CR12] Barnett J (2003). Security and climate change. Global Environmental Change.

[CR13] Bettini G (2014). Climate migration as an adaptation strategy: De-securitizing climate-induced migration or making the unruly governable?. Critical Studies on Security.

[CR14] Bhabha HK (1994). The location of culture.

[CR15] Bhatta GD, Aggarwal PK, Poudel S, Belgrave DA (2015). Climate-induced migration in South Asia: Migration decisions and the gender dimensions of adverse climatic events. Journal of Rural and Community Development.

[CR16] Black R, Collyer M (2014). Populations ‘trapped’ at times of crisis. Forced Migration Review.

[CR17] Black R, Collyer M, Martin SF, Weerasinghe S, Taylor A (2014). “Trapped” Populations: Limits on mobility at time of crisis. Humanitarian crises and migration.

[CR18] Black R, Adger WN, Arnell NW, Dercon S, Geddes A, Thomas D (2011). The effect of environmental change on human migration. Global Environmental Change.

[CR19] Black R, Bennett SRG, Thomas SM, Beddington JR (2011). Climate change: Migration as adaptation. Nature.

[CR20] Black R, Arnell NW, Adger WN, Thomas D, Geddes A (2013). Migration, immobility and displacement outcomes following extreme events. Environmental Science & Policy.

[CR21] Blaikie P, Cannon T, Davis I, Wisner B (1994). At risk: Natural hazards, people’s vulnerability and disasters.

[CR22] Bogardi J, Warner K (2009). Here comes the flood. Nature Report Climate Change.

[CR23] Bourdieu P, Wacquant LJD (1992). An invitation to reflexive sociology.

[CR24] Boykoff MT, Boykoff JM (2007). Climate change and journalistic norms: A case-study of US mass-media coverage. Geoforum.

[CR25] Bronen R (2014). Choice and necessity: Relocations in the Arctic and South Pacific. Forced Migration Review.

[CR100] Brown L, Mcgrath P, Stokes B (1976). Twenty two dimensions of the population problem. Worldwatch Paper 5.

[CR26] Cannon T, Müller-Mahn D (2010). Vulnerability, resilience and development discourses in context of climate change. Natural Hazards.

[CR27] Carling J (2002). Migration in the age of involuntary immobility: Theoretical reflections and Cape Verdean experiences. Journal of Ethnic and Migration Studies.

[CR28] Carvalho A (2005). Representing the politics of the greenhouse effect. Critical Discourse Studies.

[CR29] Castles S (2003). Towards a sociology of forced migration and social transformation. Sociology.

[CR500] Chambers R (2016). Participatory Workshop: Personal attitudes, behaviours and mind-sets, and how to transform institutional cultures. Organised on the 6th of March.

[CR30] Collyer M (2006). Migrants, migration and the security paradigm: Constraints and opportunities. Mediterranean Politics.

[CR31] Dreher T, Voyer M (2015). Climate refugees or migrants? Contesting media frames on climate justice in the Pacific. Environmental Communication.

[CR32] Dun O (2011). Migration and displacement triggered by floods in the Mekong Delta. International Migration.

[CR101] Dun OV, Gemenne F (2008). Defining ‘environmental migration’. Forced Migration Review.

[CR33] Durkheim E (1899). Friederich Ratzel: Anthropogeographie: Un compte-renduu [Anthropogeography: A review]. L’Annee Sociologique 1898–1899.

[CR34] El-Hinnawi E (1985). Environmental refugees.

[CR35] Elliott JR, Pais J (2006). Race, class, and Hurricane Katrina: Social differences in human responses to disaster. Social Science Research.

[CR36] Eriksen SH, Nightingale AJ, Eakin H (2015). Reframing adaptation: The political nature of climate change adaptation. Global Environmental Change.

[CR37] Fairclough N (1995). Critical discourse analysis.

[CR38] Fairclough N (2003). Analysing Discourse: Textual analysis for social research.

[CR39] Felli R, Castree N (2012). Neoliberalising adaptation to environmental change: Foresight or foreclosure?. Environment and Planning A: International journal of urban and regional research.

[CR40] Folke C, Carpenter S, Elmqvist T, Gunderson L, Holling SC, Walker B (2002). Resilience and sustainable development: Building adaptive capacity in a world of transformations. Ambio.

[CR41] Foresight. 2011. Migration and global environmental change: Future challenges and opportunities. Final Project Report. London: The Government Office of Science.

[CR42] Foucault, M. 1981. The order of discourse. In *Untying the text: A post*-*structuralist reader*, trans. I. McLeod. London: Routledge.

[CR43] Foucault M (2002). The order of things: An archaeology of the human sciences.

[CR44] Gale P (2004). The refugee crisis and fear: Populist politics and media discourse. Journal of Sociology.

[CR45] Gardner PD (2017). Worlds Apart: A comparative analysis of discourses of English in the curricula of England and Australia. English in Education.

[CR46] Geddes A (2015). Governing migration from a distance: Interactions between climate, migration, and security in the South Mediterranean. European Security.

[CR47] Geddes A, Adger WN, Arnell NW, Black R, Thomas DSG (2012). Migration, environmental change, and the ‘challenges of governance’. Environment and Planning C: Government and Policy.

[CR48] Gill N (2010). ‘Environmental refugees’: Key debates and the contributions of geographers. Geography Compass.

[CR49] Gray C, Wise E (2016). Country-specific effects of climate variability on human migration. Climatic Change.

[CR50] Hansen A, Oliver-Smith A, Hansen A, Oliver-Smith A (1982). Involuntary migration and resettlement: Causes and contexts. Involuntary migration and resettlement: The problems and responses of dislocated people.

[CR51] Hartmann B (2010). Rethinking climate refugees and climate conflict: Rhetoric, reality and the politics of policy discourse. Journal of International Development.

[CR52] Hess JJ, Malilay JN, Parkinson AJ (2008). Climate change: The importance of place. American Journal of Preventive Medicine.

[CR53] Hillmann F, Ziegelmayer U (2016). Environmental change and migration in coastal regions: examples from Ghana and Indonesia. DIE ERDE—Journal of the Geographical Society of Berlin.

[CR54] Hulme M (2011). Reducing the future to climate: A story of climate determinism and reductionism. Osiris.

[CR55] Humble AT (2014). The rise of trapped populations. Forced Migration Review.

[CR56] Hunter LM, Luna JK, Norton RM (2015). Environmental dimensions of migration. Annual Review of Sociology.

[CR57] Hyndman J (2012). The geopolitics of migration and mobility. Geopolitics.

[CR58] Iftekhar A, Darryn M (2014). Post-tsunami resettlement in Sri Lanka and India: Site planning, infrastructure and services. International Journal of Disaster Resilience in the Built Environment.

[CR59] Jorgensen R (2015). Language, culture and access to mathematics: A case of one remote Aboriginal community. Intercultural Education.

[CR60] Kelman I (2015). Difficult decisions: Migration from small island developing states under climate change. Earth’s Future.

[CR61] KhosraviNik M (2010). The representation of refugees, asylum seekers and immigrants in British newspapers: A critical discourse analysis. Journal of Language and Politics.

[CR62] Kibreab G (1997). Environmental causes and impact of refugee movements: A critique of the current debate. Disasters.

[CR63] Kniveton D, Smith C, Wood S (2011). Agent-based model simulations of future changes in migration flows for Burkina Faso. Global Environmental Change.

[CR64] Lister M (2014). Climate change refugees. Critical Review of International Social and Political Philosophy.

[CR65] Lu M (1999). Do people move when they say they will? Inconsistencies in individual migration behavior. Population and Environment.

[CR66] Lubkemann SC (2008). Involuntary immobility: On a theoretical invisibility in forced migration studies. Journal of Refugee Studies.

[CR67] Maldonado JK, Shearer C, Bronen R, Peterson K, Lazrus H (2013). The impact of climate change on tribal communities in the US: Displacement, relocation, and human rights. Climatic Change.

[CR68] McNamara KE (2007). Conceptualizing discourses on environmental refugees at the United Nations. Population and Environment.

[CR69] Methmann C, Oels A (2015). From ‘fearing’ to ‘empowering’ climate refugees: Governing climate-induced migration in the name of resilience. Security Dialogue.

[CR70] Milan A, Ruano S (2014). Rainfall variability, food insecurity and migration in Cabricán, Guatemala. Climate and Development.

[CR71] Morrissey J (2012). Rethinking the ‘debate on environmental refugees’: From ‘maximalists and minimalists’ to ‘proponents and critics’. Journal of Political Ecology.

[CR72] Myers N (1997). Environmental refugees. Population and Environment.

[CR73] Pelling M (2010). Adaptation to climate change: From resilience to transformation.

[CR74] Penning-Rowsell EC, Sultana P, Thompson PM (2013). The ‘last resort’? Population movement in response to climate-related hazards in Bangladesh. Environmental Science & Policy.

[CR75] Piguet E (2013). From “Primitive migration” to “climate refugees”: The curious fate of the natural environment in migration studies. Annals of the Association of American Geographers.

[CR76] Renaud, F.G., J.J. Bogardi, O. Dun, and K. Warner. 2007. Control, adapt or flee: How to face environmental migration? *UNU*-*EHS InterSecTions No. 5/2007*. Bonn: UNU-EHS.

[CR77] Reuveny R (2007). Climate change-induced migration and violent conflict. Political Geography.

[CR78] Said EW (1978). Orientalism.

[CR79] Said EW (1990). Narrative, geography and interpretation. New Left Review.

[CR80] Sherbinin A, Castro M, Gemenne F, Cernea MM, Adamo S, Fearnside PM, Krieger G, Lahmani S, Oliver-Smith A, Pankhurst A, Scudder T (2011). Preparing for resettlement associated with climate change. Science.

[CR81] Smith PJ (2007). Climate change, mass migration and the military response. Orbis.

[CR82] Sow P, Marmer E, Scheffran J (2015). Between the heat and the hardships. Climate change and mixed migration flows in Morocco. Migration and Development.

[CR83] Stal M (2011). Flooding and relocation: The Zambezi River Valley in Mozambique. International Migration.

[CR84] Stein RM, Dueñas-Osorio L, Subramanian D (2010). Who evacuates when hurricanes approach? The role of risk, information, and location. Social Science Quarterly.

[CR85] Thiede BC, Brown DL (2013). Hurricane Katrina: Who stayed and why?. Population Research and Policy Review.

[CR86] UNFCCC. 2011. Report of the Conference of the Parties on its sixteenth session, held in Cancun from 29 November to 10 December. Decisions adopted/CP.2010/7/Add. 1. Bonn: UNFCCC.

[CR87] UNFCCC. 2015. Adoption of the Paris Agreement. Draft decision-/CP.21. Geneva: United Nations Office at Geneva.

[CR102] UNHCR. 1967. Protocol relating to the Status of Refugees. In *A/RES/2198*. New York and Geneva: UNGA and UNHCR.

[CR88] Wagner M (1873). The Darwinian theory and the law of the migration of organisms.

[CR89] Warner K, Afifi T (2014). Where the rain falls: Evidence from 8 countries on how vulnerable households use migration to manage the risk of rainfall variability and food insecurity. Climate and Development.

[CR90] Weiner M (1992). Security, stability, and international migration. International Security.

[CR91] Wodak R (2011). Complex texts: Analysing understanding, explaining and interpreting meanings. Discourse Studies.

