# Supplementary material for: A discursive review of the textual use of ‘trapped’ in environmental migration studies: The conceptual birth and troubled teenage years of trapped populations
Source: Ambio. 2018 Feb 12;47(5):557–73. doi: 10.1007/s13280-017-1007-6 (PMC6072639; doi:10.1007/s13280-017-1007-6)
Supplement: Supplementary file 1 — Supplementary material 1 (PDF 184 kb) [file 13280_2017_1007_MOESM1_ESM.pdf]

Ambio

Electronic Supplementary Material

This supplementary material has not been peer reviewed.

Title: **The conceptual birth and troubled teenage years of Trapped Populations: A discursive review of the textual use of ‘trapped’ in environmental migration studies**

Authors: Sonja Ayeb-Karlsson, Christopher D. Smith, Dominic Kniveton

Extract 2

- *The complex interactions of drivers can lead to different* ***outcomes****, which* ***include migration and displacement****.* In turn, these types of outcomes can pose **more 'operational' challenges** or **more 'geopolitical' challenges**. There are **powerful linkages between them**. **Planned** and **well-managed migration** (which **poses operational challenges**) can **reduce** the **chance of** later **humanitarian emergencies and displacement**.

- ***Environmental change*** *is equally* ***likely*** *to* ***make migration less possible*** *as* ***more probable****.* This is because **migration is expensive** and **requires** forms of **capital**, **yet populations** who experience the **impacts of environmental change** may see a **reduction** in the **very capital required** to enable a **move**.

- *Consequently, in the* ***decades ahead, millions of people will*** *be unable to move away from locations in which they are extremely vulnerable to environmental change.* To the **international community**, this '*trapped*' population is likely to represent just as important a **policy concern** as those who do migrate. **Planned and well-managed migration** can be one important **solution** for this **population of concern**.

(Foresight 2011:9)

Extract 3

Cities in low-income countries are a particular concern*, and are faced with a 'double jeopardy'* ***future****.* **Cities** are likely to **grow in size**, partly because of **rural–urban migration** trends, whilst also being increasingly threatened by global environmental change. These **future threats will** add to existing **fragilities**, whilst **new urban migrants** are, and **will** continue to be, particularly vulnerable. Yet this report **argues against trying to prevent rural–urban migration,** as this could lead to **graver outcomes** for those who are **trapped in vulnerable rural areas**.

(Foresight 2011:10)

Extract 4

In summary, the ***key message of this report is that migration in the face of global environmental change may not be just part of the 'problem' but can also be part of the solution****. In particular,* ***planned*** *and* ***facilitated*** *approaches to* ***human migration*** *can* ***ease people out*** *of situations of* ***vulnerability****. In light of this,* ***international policy makers should consider*** *the* ***detailed evidence from*** *this* ***report*** *in a range of areas, with the following of particular priority:*

1. **Many** of the **funding mechanisms for adaptation** to environmental change are currently under discussion. It is imperative that these mechanisms are **not developed in isolation from migration** issues and, furthermore, that the **transformational opportunities of migration** is **recognised**.
2. Whilst the **twin challenges of population growth and environmental change will pose** an **increasing threat** to **urban areas** in the **future**, **cities in many countries** are **already failing their citizens**. **Action** is **required before** the **situation becomes irreversible**, to **build urban infrastructure** that is sustainable, flexible and inclusive.

The cost of inaction is likely to be higher than the costs of measures discussed *in this report, especially if they reduce the likelihood of* ***problematic displacement****.* ***Giving urgent policy attention to migration in the context of environmental change now will prevent a much worse and more costly situation in the future****.*

(Foresight 2011:10)

Extract 5

Proactively facilitated and **managed** migration should lead to **improvements** in each of the **future scenarios**, as it **will reduce** the chances of populations being trapped and/or being displaced in circumstances which **raise wider geopolitical challenges**. A **proactive approach** can also **capitalise** on and **maximise** the **benefits** from migration, **building resilience and transforming adaptive capacity**.

(Foresight 2011:17)

Extract 6

Reduced options for migration, combined with incomes threatened by environmental change, mean that people are likely to migrate in illegal, irregular, unsafe, exploited or unplanned ways. People are also likely to find themselves migrating to areas of high environmental risk, such as low-lying urban areas in mega-deltas or slums in water-insecure expanding cities.

(Foresight 2011:13)

Extract 7

Those with **lower wealth** or **capital** face a double set of risks from future environmental change: their **reduced level of capital** means that they are **unable to move away** from situations of increasing environmental threats; yet, at the same time, this very **lack of capital** makes them even more vulnerable to environmental change. These populations are likely to become **trapped** in places where they are vulnerable to environmental change.

(Foresight 2011:14).

Extract 8

Migration in the **context of environmental change** is likely to **lead to increased rural–urban migration** and **city expansion**. **Cities will face a 'double jeopardy' future**, in which **this challenge** is **multiplied by increasing threats from environmental change**. Yet the **third challenge** is **perhaps** the **most critical**, the **fate of the new migrant arrival to the city**, who **will** often be in the most **vulnerable situation**.

Cities will face compound **future challenges**, which **will** reinforce each other or '**multiply**' the **consequences**. These **challenges** are:

1. ***Cities are growing in terms of their populations as a result of natural population growth and increased rural urban migration***. For example, Dhaka's **population increased from 1.4 million** in 1970 **to 14 million** in 2010, and is **expected to rise to 21 million** in 2025; similarly, Shanghai's **population increased from** just over **6 million** in 1970 to **over 16 million** in 2010 and is **expected to rise** to just **over 20 million** in 2025. In a 'business as usual' scenario this **expansion alone** would **represent** a **huge set of operational challenges for cities**, including housing provision and land-use planning, **particularly** for those in **low-income countries**.
2. ***Cities are extremely vulnerable to future environmental change, particularly those located in vulnerable areas****, such as drylands, low-elevation coastal zones or mountain regions, where inundation, reduced availability of water resources and threats to health* ***will*** *variously be experienced*. For example, the **populations** living **in urban floodplains** in Asia may ris**e from 30 million** in 2000 **to between 83 and 91 million** in 2030, and **then to 119–188 million** in 2060 according to **different scenarios of** the **future**. The **future expansion of cities needs to be understood in the context of this increasing risk**.

(Foresight 2011:19).

Extract 9

Policies to avoid *populations being trapped in* ***conflict*** *situations, where they are in turn vulnerable to environmental change*. Where there is an **endogenous and cyclical relationship** between **poverty**, **resources**, **conflict** and the inability for people to move voluntarily (with humanitarian emergencies and displacement a likely outcome), an important set of **policies** should focus on **reducing conflict** and **tension** associated with **natural resources**. Environmental change is likely to affect these **natural resources**, potentially reinforcing this endogenous cycle; there is thus a clear requirement for **policies to address** the impact of environmental change on the **resource–conflict relationship**.

(Foresight 2011:21)

Extract 10

Conflict and **poverty** are two **contributors to vulnerability within 'poor and high-risk environments'**, which include drylands[211]. **Although** there is **disagreement** as to **whether**

environmental change leads to conflict[212], it is **clear that communities** which are **subject** to **increasing environmental variability** and **disruption** are **likely** to **become poorer**. The important point is that **poverty lessens** their **ability** to **respond** in a **planned** and **controlled way to threats**, whether they be **ecological**, **conflict related**, **economic** or **demographic** (prevalence of disease). This **includes planned migration**, which is **often** an **appropriate response to these threats**, but likely to be **curtailed** by **low capital** (social, political or **economic**) and **conflict**.

(Foresight 2011:73)

Extract 11

As noted in section 9.3.6, the **timing for actions relating** to adaptation **funding** is **important**. The **urgency** of the issue **in respect to cities** requires particular emphasis. Whilst trends of **global environmental change and population growth** are **likely to multiply the challenges faced by cities in** the **future**, it is important to recognise that these **challenges will** add to *existing* **fragilities**. **Many cities in low-income countries** are **already failing** in important respects, and **citizens**, **especially low-income groups** such as **migrants**, are **already extremely vulnerable**. **Future trends** are set to **exacerbate** these **challenges**, and **action** is **required before** it is **too late**.

(Foresight 2011:198-199)

Extract 12

The analysis also highlights that low mobility is **critical**— that **populations** may be, in effect, trapped in places where environmental **risks** are increasing (**Black** et al 2013). The **Foresight analysis demonstrates** how **migration** is a well-documented and often **effective adaptation** to environmental **risks** (**Black** et al 2011b).

(Adger et al. 2015:3)

Extract 13

Individual disasters and events also **negative economic impacts**—Hallegatte (2012) emphasizes the prospect of **people losing their assets** and **falling into poverty traps**. **A lack of capital** at the individual level is, indeed, **one of the principal causes of immobility** and potentially trapped populations (**Black** et al 2013).

(Adger et al. 2015:4)

Extract 14

However, without minimising the significance of the ‘right to stay’ even in places that are vulnerable to environmental extremes, it is also clear that **ability to move** is broadly **correlated with wealth**, **level of capital (financial, human, social),** the availability of places to move to, and **fear of what would happen to property and assets left behind**, so that broadly speaking, **poorer people are generally less able to migrate** even if they wish to do so. In turn, **vulnerability** to extreme environmental events is **widely recognised to be inversely correlated with wealth**, such that **poorer people face a double risk**: they are more vulnerable to disasters, but less able to move away from them. This lack of choice for vulnerable populations is recognised in both behavioural accounts of vulnerability and by the pressure-and-release structural models of vulnerability (see Wisner et al., 2004). Fig. 3 therefore depicts **the two-dimensional space for populations** where **mobility potential and wealth are generally positively** correlated and where **vulnerability to stress is inversely correlated to wealth for individuals**. **Trapped populations** are **vulnerable** to stress but **without the ability or resources** to move.

(Black et al 2013:S36)

Extract 15

Similarly, **worse-off households** might be prevented from sending migrants following shocks to their **income** and **assets**, thus representing **“trapped populations”** (**Black et al**. 2011).

(Gray and Wise 2016:556)

Extract 16

The **Foresight report on Migration and Global Environmental Change** shed light on two relatively understudied issues in the literature on climate change and migration. First, it emphasized the importance of studying the specificities of mountain areas in order to understand the nexus between environmental change and migration in those areas (Kollmair & Banerjee, 2011). Second, it showed that **future environmental change** is equally **likely to lead** to an increase or a decrease in migration flows. In this context, those who might be willing but unable to move **("trapped") will** be extremely **vulnerable** (**Foresight**, 2011). However, the **Foresight report** did only refer to few empirical studies on **trapped populations**. This empirical article aims at presenting data and insights on four Guatemalan mountain **communities** whose **populations** are exposed to the **risk of becoming "trapped" in the near future** in a place where they are extremely vulnerable to climate change. In fact, **in case of future natural disasters** or climatic conditions which **threaten the sustainability of local livelihoods**, it is expected that **migration will be vital for the survival of these populations**.

(Milan and Ruano 2014:61-62)

Extract 17

The review above gives details of the changing hazardousness of Bangladesh; what we see is a decline in mortality over time but a **continuation of substantial economic losses** and in some cases a **substantial threat to food supplies**. What, then, are the related migratory effects? Have those parts of the population who have suffered from these disasters – and **will no doubt suffer again in the future** – sought to move away from the areas affected, or are they in some way **''immobile''** or **''trapped''** where they currently live (**Foresight**, 2011)?

(Penning-Rowsell et al. 2013:S49)

Extract 18

For example, in the case of Bangladesh, we have the outliers of **households** that do not even need to migrate, as they are already well off, whereas there are other **households** in **extreme poverty** and **vulnerability**, for which migration is not even an option, as they **lack the means** to move and are forced to stay '**trapped'** *in situ*.

(Afifi et al. 2015:13)

Extract 19

In Vietnam, **HHs** that **suffer from poverty** and **do not benefit from the economic boom** are **often left behind (trapped populations**).

(Warner and Afifi 2014:7)

Extract 20

Migration is a major **risk management**/'coping strategy' to **address unfavourable economic** and unexpected environmental conditions, including the local implications of rainfall variability. Longer dry spells and frequent droughts are a 'very important' migration reason for 39% and 36% of **HHs**, respectively. **Landless**, **low-skilled** and **poor HHs** (depending on rain-fed agriculture for both their **livelihoods** and **food security**) are the most sensitive to rainfall variability. Also often **trapped due to lack of resources**.

(Warner and Afifi 2014:8)

Extract 21

The final profile of **HHs** includes those that have been described as **'trapped populations'** in the literature: **HHs** that **do not possess the assets** necessary to migrate, even to cope with **food insecurity**, or who cannot access migration options. These are often **landless or land scarce HHs** in very **poor areas**. /…/ **For trapped HHs or populations**, repeated environmental shocks and stressors can continue to **erode their asset base and increase their food and livelihood insecurity**.

(Warner and Afifi 2014:13)

Extract 22
We suggest there are six major mobility outcomes:
(1) migration within **states**;
(2) migration between **states**;
(3) displacement within **states**;
(4) displacement between **states**;
(5) choosing not to move;
(6) being unable to move and **trapped**.

These outcomes cannot be understood simply as ex post **challenges to governance systems**. In the next section we develop a conceptual **understanding of governance** and then develop this insight to show how migration is constituted as a **governance challenge** by the effects of and interactions between five social and natural systems (economic, social, political, demographic, and environmental) that drive migration and also determine whether or not people move, as well as the scale, direction, and duration of movement.

(Geddes et al. 2012:953)

Extract 23
Tens of millions of people may find themselves **trapped** in vulnerable areas and unable to migrate.

(Geddes et al. 2012:962)

Extract 24
Thus the resultant **challenges** may be **more complex than first thought** and hold important **implications for SMPCs and for the EU**. First, **movement** may be **towards and not away from risk**, **particularly for economic reasons to large cities**. Second, environmental **change** may interact with other factors to reduce the ability to migrate and can lead to **situations where people are trapped** in **areas** in which they are **exposed to serious environmental risk**.

(Geddes 2015:488-489)

Extract 25
Political **upheaval** has been a key migration **driver** in SMPCs. **Conflicts** such as the **Gulf Wars** and in **Israel**, **Palestine**, **Libya**, and **Syria** have all led to **massive displacement**. **Conflict** can also cause **people to be trapped** in areas rather than for them to be displaced, thus making **conflict-related movements** particularly **unpredictable**, **dynamic**, and **hard to analyse**.

(Geddes 2015:481)

Extract 26
As **border security increases** and **borders become less permeable**, **cross-border migration** is becoming **increasingly difficult**, **selective** and **dangerous**. **Growing numbers of people** are becoming **trapped in their own countries or in transit countries**, or being **forced to roam border areas**, **unable to access legal protection** or **basic social necessities**.

(Humble 2014:56)

Extract 27
Even if migrants' circumstances fall within legal protection frameworks, **strict border controls** mean they often **cannot access protection** and are **trapped** on the **'wrong side' of the border**. /.../ There are many **hotspots** where **concentrated groups of people** become **trapped due to border security** – such as in northern **France**, north-west **Turkey**, northern **Bangladesh** and **North Korea** – often congregating in **informal 'migrant camps'**, with **many similar scenarios worldwide**.

(Humble 2014:56)

Extract 28

These **trapped migrants** are **vulnerable**, **exposed to** the **violations** and **abuses** that are typical for those moving through countries irregularly, including: **not having access to basic necessities**; **discrimination** and **abuse** because of their **foreign origin** and **irregular status**; **human trafficking** (which exposes migrants to **coercion**, **deception** and **physical and sexual abuse**); **dangerous** or **forced labour**; and **organ theft**.

(Humble 2014:56)

Extract 29

The **transitory settlement** of **West Africans in Moroccan cities** has led to noticeable **changes** in the appropriation and **degradation of spaces and places** in the **absence of interventions by the Moroccan government to offer legal protection** and **institutional support** for most of **African immigrants**, while **Europe increases** its **measures to prevent them from entering**. **West African immigrants** become **trapped in this situation** and most often **experience hostility, racism and violence**.

(Sow et al. 2015:1)

Extract 30

To be '**trapped**', **individuals** must **not only lack** the **ability to move** but also either **want or need to move**. The **ability to migrate** is clearly a **complex** and **multifaceted** indicator that includes a **range of potentially relevant policies** that may **impede movement** and **access to significant resources**.

(Black and Collyer 2014a:52)

Extract 31

To be **trapped**, **individuals** must **not only lack** the **ability to move** but also either **want or need to move**. **Ability to migrate** is clearly a **complex** and **multi-faceted** indicator that includes **access to significant resources** or **capitals** and **a range of potentially relevant policies** that may **impede movement**.

(Black and Collyer 2014b:298)

Extract 32

A striking **example** is New Orleans at the time of **Hurricane Katrina**. **Those with resources left in advance** of the approaching hurricane; those with **friends and family elsewhere**, with whom they could **go and stay**, were also more likely to leave. Those without resources (largely the poor, **African-American**, **elderly** or residents without private cars) remained, **trapped** as the floodwaters rose. The **dangers** of the crisis were **disproportionately** faced by the **most vulnerable**. Where mobility brings benefits, **trapped populations** are further **marginalised**.

(Black and Collyer 2014a:54)

Extract 33

It is **not necessary** for **trapped individuals** to have **always remained in one place**. **Conditions** that **trap** particular populations may arise at **any stage in their migration process**. Protracted **refugee situations** offer an obvious example of a **partially mobile yet trapped population**.

(Black and Collyer 2014b:298)

Extract 34

Thus **individuals** may ***need*** **to move**, *and* be **offered an opportunity** to do so, under **particular conditions** but **still refuse to leave**. Such **individuals must still be considered** as **trapped**.

(Black and Collyer 2014b:300)

Extract 35

There is only very **limited research investigating** the **situation** of those we have **called "trapped" populations**. **Much** of this has **focused** on immobility more **broadly**, rather than the specific difficulties of those **trapped** as a result of crisis situations. The additional consideration of "**needing**" **to move** introduced a specifically **humanitarian focus** to our **understanding** of **"trapped"**.

(Black and Collyer 2014b:300)

Extract 36
Research into the situation of those who are **trapped** in complete immobility presents the greatest difficulties. The **Foresight report considers their difficulties as largely economic**. As the **review of resource constraints** in the previous section demonstrated, this **can take many forms**; **constraints may not be directly financial** and **may include** things such as **access to geographically distant social networks**.
(Black and Collyer 2014b:301)

Extract 37
While we have such **limited information** on **trapped populations**, the **policy goal** should be to **avoid situations** in which **people are unable to move** when they want to, **not** to **promote policy** that **encourages** them to **move when they may not want to**.
(Black and Collyer 2014b:302)

Extract 38
The most **urgent issue** is to **identify how existing responses** can **reduce** the **likelihood** of **individuals** being **trapped** in crisis situations. At present **our understanding** of the mechanics of **trapped populations is too limited to suggest any clear policy measures** to **reduce** their **vulnerability or enable** them to **move when they feel they need to**.
(Black and Collyer 2014b:303)

Extract 39
Another 'spatial perspective' in the debate of migration and environmental change is put forward by ***Black*** **and** ***Collyer*** **(2014)**. The **authors claim** that today **"trapped populations"** do **exist**, people that – out of a **lack of resources or other restrictions** – are **unable to move** when confronted with an environmental shock situation.
(Hilmann and Ziegelmayer 2016:122)

Extract 40
This **situation** hints to the **existence** of a **large proportion of the population** that the **recent literature** has **classified** as **"trapped populations" (Black and Collyer, 2014)**.
(Hilmann and Ziegelmayer 2016:133)

Extract 41
In the Indonesian case **people** showed to be much more **attached to their place of residence by feelings of belonging** as well as **belief-systems**. Here the concept of **mental thresholds** as put forward by *van der Velde* and *van Naerssen* (2007b) seemed to be at work. Additionally, immobility and "**trapped populations**" **stood out** as a constitutional part of the nexus between environmental change and migration.
(Hilmann and Ziegelmayer 2016:133)

Extract 42
Households that **cannot migrate**: The third group includes **trapped populations** that struggle to survive under adversity and cannot easily use migration to adapt to the negative impacts of climatic events. They may have **strong social ties** and are **emotionally attached** to their **resources** which **restrain** them **from moving even under adverse events**.
(Bhatta et al. 2015:14)

Extract 43
The **socio-economic**, **cultural** and **religious values** in the region have resulted in its **women** and **children** being **more vulnerable** to adverse situations in comparison to men. These **dynamics form** the **vicious cycles where women and their children get trapped**.
(Bhatta et al. 2015:15)

Extract 44
The trapped group has **intermediate income including women who are generally less likely to move**, due to **socio-cultural and economic reasons**, as are **children** and the **elderly**, and therefore **local adaptation efforts should target these groups specifically**. Given that **men's migration could contribute** to the **further deprivation and risk that women face**, the **trapped population needs** to be **provided with greater social protection** and **opportunities** for livelihood security.

(Bhatta et al. 2015:17-18)

Extract 45
This research focuses on the intermediate stage of migration decision-making between experiencing stress and migrating and **seeks to determine what stops a person from relocating**, even **when** they are **dissatisfied**. Therefore, **this article brings behaviourist ideas to the trapped populations thesis** in order **to create a more diverse conceptualisation of trapped** in the context of immobility. **The article builds on insights from social and behavioural theories** of migration decision-making (e.g. Speare 1974) and **ideas of place attachment** and **social capital**, applying these to the context of **populations** that **decide to remain in location**, **despite** exhibiting **dissatisfaction with their present location** and living under difficult environmental conditions.

(Adams 2016:431)

Extract 46
An understanding of the highly differentiated nature of mobility, **residential satisfaction and place attachment illuminates** some of the **reasons why rural populations** continue to **persist across the globe**, even in the face of **difficult conditions**. **This expanded view of what it means to be "trapped" suggests** that **such rural populations will continue to persist** even under a climate-changed future.

(Adams 2016:445)

Extract 47
Material as well as **subjective dimensions of the decision to migrate** (both in the **ways people experience impacts** (e.g. Massey et al. 2010) and their **capacity to respond** through migration) is likely to be **highly differentiated across** the **population**, **yet authors discuss a single** "**trapped**" **population**.

(Adams 2016:431)

Extract 48
I would argue that **this form of immobility** can be **interpreted as** the **individual being "trapped**". **This contrasts** with the **current definition of trapped populations** as **people** or **households without** the **means to escape a dangerous natural disaster**, **for example** those **without transport** who could not leave New Orleans **during Hurricane Katrina** or **without the resources to overcome barriers during agricultural decline** or **environmental degradation** (**Warner and Afifi** 2014).

(Adams 2016:443)

Extract 49
Therefore, **just as it is accepted** that migration under environmental change exists on a continuum from forced to voluntary (Hunter 2005), **a similar continuum exists for trapped populations**. At one end, is **the traditional definition of trapped**, where **people are physically unable to leave** a **risky location** and their **lives** are **under threat**.

(Adams 2016:443)

Extract 50
Common responses were: ''Lack of money, everything is money in Lima, and **if you don't have a property you've got to pay to rent a place''** or **''I don't have a house, nor work [in Lima], that's why I don't go''**. This **group most closely fits** the **description of "trapped**" which is currently used in the environmental migration world: **low income households** that are **not able to use migration** as a

strategy **to improve well-being for their family** (Box 2 in Fig. 2).

(Adams 2016:440)

Extract 51

Of **those who** have **experienced dissatisfaction with location**, **only 26 % are "trapped" by the current definition**, that is to say, **unable to migrate because of resource barriers**. Even then, these **responses may reflect a low perceived self-efficacy** on the part of the potential migrant, **rather than** an actual **lack of resources** to migrate. **Forty per cent of the population is in location because of social and affective attachment to place**, "**trapped" by their own internal attachment or fear of the outside world**. **This represents 74 % of the dissatisfied population**.

(Adams 2016:442)

Extract 52

Not to put too fine a point on it, the **promotion of migration as adaptation strategy** is **consistent** with the **neoliberal practice of constituting a new global reserve army of labour** (Taylor, 2009). Indeed, the **promotion of migration as adaptation** is **not only justified with** regard to the fate of **`trapped'** or **poor populations**, but is also **presented** as **having important benefits for** the **receiving states**. The report is **not advocating a policy of open borders but**, instead, one in which **migrations are encouraged** as well as **monitored and managed**. This is especially the case with the seemingly **uncritical promotion of ``temporary and circular migration schemes**''.

(Felli and Castree 2012:3)

Extract 53

In this last part the **report encourages policy makers** to **promote migrations** (both **internal** and **international**) that can **benefit** both potential and actual **migrants by allowing them to 'escape' areas** that are **suffering** adverse **environmental change** (with a **special focus** on '**trapped' populations'**), to bring **developmental benefits for their territory** or **community** of origin (notably **through remittances**), and to have a **positive impact** on the **countries of destination** [by **introducing** a **younger** and more **entrepreneurial workforce than** the **domestic** on].

(Felli and Castree 2012:2)

Extract 54

Obstacles to migration remain extremely important, and **large vulnerable populations remain trapped in** highly **vulnerable regions**. **In the absence of a global solution**, it is likely that most **policy responses** will **remain regional** and **humanitarian** in nature.

(Baldwin and Gemenne 2013:265)

Extract 55

But for our purposes, **the Foresight Report is important because it functions as a** kind of **security apparatus**; it **installs an affective infrastructure** through which **climate change** comes to be **conceived as a problem of racial management**, albeit in a way that **obscures** any obvious **racial connotations**. The **text advances the desirability of adaptive migration**, the **benefits** of **which ought to be 'maximised'**. But it also **stipulates** that **maladaptive migration** (migration into areas of high environmental, **social** and **political risk**, such as **urban informal settlers**; and **so-called trapped or immobile populations**) **requires new forms of intervention**. **Maximising adaptive migration in the interest of capital circulation** and **planetary well-being**, **while containing maladaptive migration** bears **striking resemblance to Foucault's biopolitical formulation 'making live and letting die'**. And as we saw earlier, this is **an** unmistakably **racial formation – a 'break into the domain of life that is under power's control'**.

(Baldwin 2016:87)
